# A Case of Continuous Negative Pressure Wound Therapy for Abdominal Infected Lymphocele after Kidney Transplantation

**DOI:** 10.1155/2014/742161

**Published:** 2014-10-08

**Authors:** Marco Franchin, Matteo Tozzi, Gabriele Soldini, Gabriele Piffaretti

**Affiliations:** ^1^Vascular Surgery, Department of Surgery and Morphological Sciences, Insubria University School of Medicine, Circolo University Teaching Hospital, Via Guicciardini 9, 21100 Varese, Italy; ^2^Center for Research on Organ Transplantation, Insubria University School of Medicine, Circolo University Teaching Hospital, 21100 Varese, Italy; ^3^General Surgery 1, Department of Surgery and Morphological Sciences, Insubria University School of Medicine, Circolo University Teaching Hospital, 21100 Varese, Italy

## Abstract

Lymphocele is a common complication after kidney transplantation. Although superinfection is a rare event, it generates a difficult management problem; generally, open surgical drainage is the preferred method of treatment but it may lead to complicated postoperative course and prolonged healing time. Negative pressure wound therapy showed promising outcomes in various surgical disciplines and settings. We present a case of an abdominal infected lymphocele after kidney transplantation managed with open surgery and negative pressure wound therapy.

## 1. Introduction

Lymphocele is a common complication after kidney transplantation [1–3]. Although it is a rare event, superinfection may complicate the postoperative course or even threaten survival [4, 5]. It also generates a difficult management problem whose resolution is not made easier by the lack of literature data [6]. Up to now, infected lymphocele has been preferentially treated with open surgical drainage; however, these wounds often caused subsequent complications requiring prolonged healing time [5, 7]. Moreover, it increases the financial burden in transplanted recipients [8]. Negative pressure wound therapy (NPWT) was developed to promote healing of open wounds and actually has found wide application in various surgical disciplines and settings [9–12].

The aim of this paper is to report a case of an infected lymphocele after kidney transplantation managed with surgical drainage and subsequent application of NPWT.

## 2. Case Report

**Figure 1:**
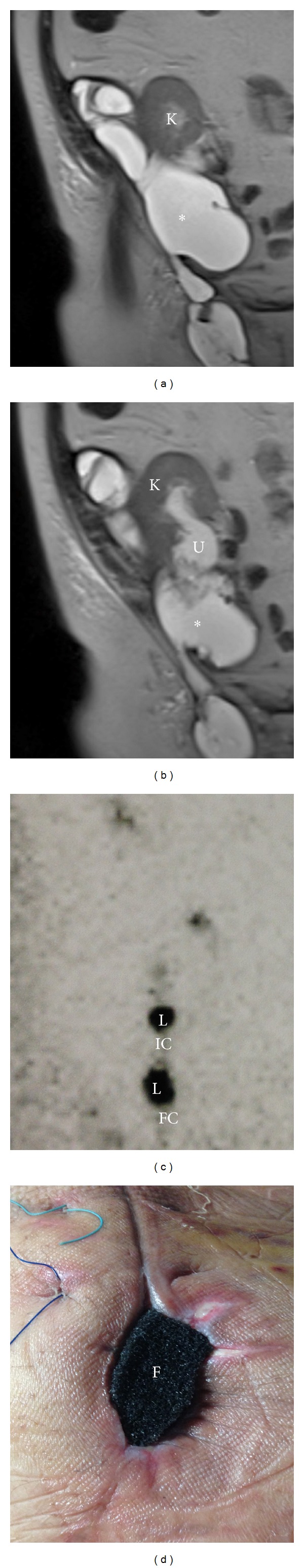


He is a 65-year-old male, Rh positive group B, with a medical history significant for hypertension, dyslipidemia, obesity (body mass index = 32), chronic obstructive pulmonary disease, ischemic heart disease treated with percutaneous transluminal coronary angioplasty, and end-stage renal disease due to glomerulonephritis. Kidney transplantation was performed using a retroperitoneal approach on common iliac vessels, while ureteral implantation was accomplished with the Lich-Gregoir technique. Kidney donor was a Rh positive group B 60-year-old male, died for cerebral hemorrhage: his serology (HBcAb, VDRL, TPHA, HIV, HCV, and HBsAg) and cultures for active infections were negative. HLA-A, HLA-B, and HLA-DR loci presented a single mismatch, evenly. Induction immunosuppressive treatment consisted of monoclonal antibodies (Basiliximab, Simulectm; Novartis, Basel; Switzerland), whereas maintenance immunosuppressive therapy included cyclosporine (Sandimun Neoral, Novartis, Basel; Switzerland) and Everlomus (Certican, Novartis, Basel; Switzerland), in association with steroid. Postoperative course was uneventful; he was discharged on day 12th with a serum creatinine level of 1.2 mg/dL. Two months after intervention a magnetic resonance angiography (MR-A) was performed accordingly to our follow-up protocol: a 150 mm × 20 mm lymph collection was detected between the graft and the abdominal wall (Figures 1(a) and 1(b)). At that time, clinical signs of infection were absent and blood tests were in normal range (C-reactive protein (CRP) = 12 mg/L, leukocytes = 8.230/mm^3^, serum creatinine = 1.17 mg/dL). It was asymptomatic; hence, we decided to continue with close renal function surveillance, delaying any percutaneous procedures. On day 46 after intervention, fever (38.5°C) and oliguria abruptly developed, with local signs of infection: blood tests showed an increase of CRP (206 mg/L), serum creatinine (2.2 mg/dL), and leukocytes count (15.220/mm^3^). Soon after, a broad spectrum antibiotic therapy with (Cefotaxime, Max Farma; Castel San Giorgio, IT) was started and urgent surgical drainage was performed. Intraoperative cultures were positives for* Escherichia coli, Staphylococcus haemolyticus,* and* Enterococcus faecalis*; therefore, we modified the antibiotic regimen adding daptomicine (Cubicin, Novartis Europharm; Horsham, UK). Systemic sepsis resolved but a 99^m^-technetium renal scan showed an abnormal lymphatic leakage in the right iliac fossa (Figure 1(c)). A change in immunosuppressive therapy was not considered. The remaining lymphatic cavity was large, scar margins were widely dehiscent and the graft exposed. We decided to use a NPWT (Renasys, Smith & Nephew; London, UK) medication to assist wound healing: the cavity was then filled with polyurethane foam dressing connected via a tubing system to a portable device using continuous 80 mmHg negative pressure. He was discharged twelve days later, on oral antibiotic therapy with amoxicillin/clavulanic acid: at that time, creatinine was 1.63 mg/dL; leukocytes count 9.300/mm^3^. This system was subsequently renewed every five days (Figure 1(d)). Forty-five days later, lymphocele was sealed and skin closed. Wound cultures were negative. Ultrasound evaluation confirmed the complete resolution of the lymphatic collection. He was last seen 6 months after the intervention asymptomatic with stable serum creatinine at 1.32 mg/dL. No ventral hernia developed.

## 3. Discussion

Lymphocele incidence rate is reported in the range from 0.6% to 49% [1–3]. This broad limit may be determined at different levels: it may be explained by the variety of lymphocele definition, as well as an underestimation in reporting results, and also because of different follow-up to which this complication has been reported [4]. Infected lymphocele is even more rarely described in literature [6]. The case report we are presenting is the only one in our experience which means an incidence rate of 0.5%. This data is consistent with the literature: out of 11.497 kidney transplantations in thirteen studies which reported the operative management of posttransplant lymphocele, we found only five papers with a total amount of 22 cases and an incidence rate of 0.2% (Table 1) [4, 5, 13–23]. We cannot assure if it happens because of low infection rate or it was an author omission; nonetheless, in these studies, information was incompletely reported (Table 2) [6]. This goes at the expense of the fact that, though loss of graft was never reported, septic complication should have been ascribed to lymphocele superinfection.

Once the diagnosis is established, uncertainty coming from these data opens the equally important debate of the best treatment for an infected lymphocele [5, 19, 23, 24]. Percutaneous drainage and/or sclerotherapy, open evacuation, and surgical fenestration into peritoneal cavity have been proposed in the past for symptomatic lymphocele: unfortunately, precise and widely adopted strategies are missing and results have been described with mixed success [5, 7, 22, 23, 25–27]. Percutaneous drainage is a minimally invasive option which does not preclude subsequent surgery; however, it has been associated with a high risk of recurrence and superinfection also because it has been used alone [6, 28]. This is the main reason why Hamza et al. [5] in their treatment algorithm advocated the use of systemic antibiotic therapy and continuous internal-external drainage in every case of suspected infection or confirmed microbiological determination. In the present case, the risk of potential iatrogenic superinfection in such a sensitive recipient led us to prefer a conservative approach. Instrumental diagnosis confirmed the nature of the collection without the need of biochemical confirmation on aspirated liquid. We agree with most of the authors that large lymphocele should be treated with open surgery [6, 19], first and foremost because it is effective on an active infection and limits the potential superinfection. Furthermore, the persistence of a lymphatic drainage or residual debris and fluid collection could be a dangerous gateway to the graft or a favourable soil for other infective agents. These latter are the main reason to take advantage of the NPWT qualities: it removes excessive or contaminated fluids and assists wound closure [9, 29, 30]. Moreover, the presence of an adhesive vapour-permeable plastic drape facilitates gas exchange, thus working against the development of anaerobic organisms and preventing access to external agents [11, 31].

Several studies have documented the appropriateness of the NPWT use in the treatment of lymphocele and lymphocutaneous fistula; however, the application of NPWT for abdominal injuries is still debated [9–13]. Accordingly to Mendez-Eastman et al. [31] the exposure of intra-abdominal organs and blood vessels is a contraindication to NPWT because of the risk of tissue damage due to subatmospheric pressure. Precise guidelines in this latter circumstance are missing: up to now, few data are available on the use of NPWT for dehiscent wounds after organ transplantation: Shrestha et al. [32] first presented a case series of 9 kidney recipients treated with NPWT for infection-related wound dehiscence. None of these patients had lymphocele or deep wounds in continuity with the iliac vessels such as in our case; nevertheless, in their cases NPWT assisted wound healing in a significantly shorter time. In similar circumstances, Heap et al. [11] treated with NPWT two complex urinary fistulas. Although literature is sporadic and heterogeneous, these reports encouraged us about the possibility to use NPWT. This is the reason why we did not change the ongoing immunosuppressive therapy: in fact, responsibility of mTOR inhibitor in the genesis of lymphocele is widely recognized [33, 34]. We have to point out that literature did not help us with practically guidelines for the application of NPWT: hence, type and intensity of negative pressure applied to the cavity were borrowed from our experience on NPWT in open abdomen [35].

In our case, major concerns were related to the fact that NPWT would have been needed for a long time even at home, which meant poor medical control in a patient with an open abdominal wound and a transplanted kidney. Surprisingly, we were impressed by the fact that this system was well tolerated by the patient: on our behalf, we established a rigorous outpatient reevaluation every three days to assess dressing integrity and efficiency of the machine. Honestly, we recognize the limitation of our case report which lends itself to have an anecdotal value. Further, this type of treatment may not be used in uncooperative or not motivated patients, and question about the financial burden of this therapy can be an obstacle to its broad application [36]. Nevertheless, it was very easy to guarantee an accurate control of either local infection or lymphatic leak. The cost-effectiveness analysis of the Medicare charges and providers confirmed a lower long-term costs when using NPWT if compared with standard wound care methods [8, 37].

## 4. Conclusion

Our case showed NPWT was effective to assist the treatment of a symptomatic infected lymphocele; it also proved to be a safe management alternative for a complex deep infected wound in a transplant recipient too. We hope our case will find further confirmation in future studies.

## Figures and Tables

**Table 1:** 14-year literature review on posttransplant lymphocele: 15 cases operatively managed at least.

Author	*N* of transplant	Lymphoceles (*n*)	Incidence (%)	Symptomatic lymphoceles (*n*)	Presentation days median (range)	Diameter (cm) mean (range)	Volume (mL) mean (range)	Graft loss (*n*)	Infection (*n*)	Conservative management *n* (recurrence)	Percutaneous treatment *n* (recurrence)	Laparoscopic fenestration *n* (recurrence)	Open surgery *n* (recurrence)	Surgical complication (*n*)
Duepree et al. [13]	1502	90	5.9	90	52 (13–1398)	ND	ND	0	0	0	0	56 (4)	34	4
Hamed et al. [12]	480	31	6.4	31	66 (19–111)	ND	200 (45–2000)	0	0	0	31 (19)	19 (4)	4	0
Bailey et al. [14]	1863	60	3.2	60	ND	ND	ND	0	0	0	0	20 (2)	42 (2)	0
Øyen et al. [15]	385	34	8.8	34	147 (18–1110)	ND	ND	0	0	34	0	26 (2)	10 (1)	0
Florian Fuller et al. [16]	138	36	26.1	36	ND	ND	480 (ND)	0	6	0	20 (6)	8	14	0
Hamza et al. [5]	620	42	6.8	42	ND	ND	250 (80–125)	0	8	0	42 (24)	17 (3)	10 (1)	0
Atray et al. [17]	1289	24	1.9	24	121 (35–631)	ND	977 (200–2000)	0	4	3 (0)	7 (5)	3	17 (3)	1
Smyth et al. [18]	386	24	6.2	13	29 (17–40)	ND	354 (ND)	0	3	0	13	0	3 (0)	0
Król et al. [19]	2147	17	7.9	17	42 (10–90)	ND	ND	0	0	0	11 (7)	0	13 (0)	0
Ulrich et al. [4]	426	42	9.9	24	ND (2–4)	7.8 (ND)	ND	0	0	18	7 (7)	26 (2)	0	0
Zargar-Shoshtari et al. [20]	744	36	4.8	14	ND (10–182)	ND	ND	0	0	ND	14 (7)	5 (1)	3 (0)	0
Lee et al. [21]	154	9	5.8	9	19 (6–28)	5 (1.5–8)	ND	0	1	5 (3)	7 (4)	1 (0)	3 (0)	0
Choudhrie et al. [22]	1363	35	2.5	35	29 (7–3670)	ND	ND	0	0	ND	25 (7)	17 (0)	0	0

*n*: number; ND: not determined.

**Table 2:** Literature data on infected lymphocele.

Author	Infection	Systemic complication	Fluid culture positivity	Treatment		Sclerotherapy	External drainage	Fenestration	
	(*n*)	(*n*)	Gram negative	Gram positive	Antibiotic first				
			*Klebsiella *spp.	*Staphylococcus *spp.	*Enterococcus *				
Florian Fuller et al. [16]	6	ND							
Hamza et al. [5]	8	0	ND	8	0	8	0		
Atray et al. [17]	4	0	ND						
Smyth et al. [18]	3	0	0	2	1	3	1	1	1
Lee et al. [21]	1	0	1	0	0	0	0	0	1

*n:* number.
